# Disaster risk reduction and Evangelical Christianity: a case for pluriversality in practice

**DOI:** 10.1111/disa.70035

**Published:** 2026-01-07

**Authors:** Scott D. Watson

**Affiliations:** ^1^ University of Victoria Canada

**Keywords:** climate change, disaster risk reduction, Evangelical Christianity, pluriversality, traditional, Indigenous and local knowledge (TILK)

## Abstract

As climate change increases the probability and intensity of major disasters, urgent action is required to prepare for and address the underlying causes of disaster. The disaster risk reduction (DRR) framework was adopted to focus national and international attention on the social production of vulnerability to disaster risk, yet it faces politically mobilised opposition, including from the Evangelical Christian (EC) community. This article suggests that DRR's engagement with traditional, Indigenous, and local knowledge is ill suited to deal with such challenges and explores the concept of pluriversality as a way to create points of interconnection and pragmatic engagement between DRR advocates and ECs. The paper argues that pluriversality provides a standard for inclusion of radical difference without embracing epistemic relativism or modernist domination. Rather than insisting on modernist terms of engagement and the rejection of religious narratives of disaster, this paper argues that recognising value in different narratives of nature and disaster is necessary to contest fatalist and scapegoating narratives that underpin EC opposition to DRR and to spur pragmatic action on DRR.

## 1 INTRODUCTION

Climate change has increased the probability and intensity of major disasters. Heat waves, floods, droughts, and wildfires are more frequent and costly, while long‐term changes in the environment are contributing to large‐scale displacements. In 2024, 152 unprecedented extreme weather events led to the highest level of internal displacement since 2008 (WMO, 2025). That same year, economic losses due to disasters reached a record high of USD 417 billion (Vanderford, 2025). Indigenous communities around the world have reported long‐term, drastic changes in weather patterns, sea and ice levels, and flora and fauna foundational to their way of life (Green and Raygorodetsky, 2010). The Intergovernmental Panel on Climate Change (IPCC)'s Sixth Assessment Report concludes that ‘[h]uman‐induced climate change’ will produce ‘more frequent and intense extreme events' and has already produced ‘irreversible impacts' that have destroyed the adaptive capacity of natural and social systems (Pörtner et al., 2022, p. 9). It predicts that by 2100, humanity will experience four to five times the number of weather‐related disasters as compared to the past (Borenstein, 2022).

For many, these findings demonstrate an urgent need to address the underlying causes of disaster and to prepare local communities for such events. The *Sendai Framework for Disaster Risk Reduction 2015–2030* (SFDRR) is the most significant global effort to do so (UNISDR, 2015). This framework reorients international and national action to address the root causes of disaster risk while preserving the market‐oriented, state‐based order (see, for example, Tozier De La Poterie and Baudoin, 2015; Gaillard, 2019; Hollis, 2020). Yet, the climate–disaster narrative that enjoys near scientific consensus and that forms the basis of disaster risk reduction (DRR) is not the only interpretation of disaster.^1^ Significant constituencies do not accept the climate–disaster narrative and comprehend disasters, climate science, state authority, and global governance frameworks in radically different ways. In many societies, these constituencies are increasingly influential and politically organised in opposition to basic tenets of DRR. One such example is the Evangelical Christian (EC) group in the United States, and EC interpretations of disaster (while varied) often support fatalist, scapegoating, or conspiratorial narratives. Strong support within the EC community for policies dismantling climate research and disaster preparedness in the US (and globally) suggests that DRR advocates need to consider urgently how to engage with radically different views of disaster.

The DRR framework already recognises the need to engage with different forms of knowledge on disaster and calls for the inclusion of traditional, Indigenous, and local knowledge (TILK) that is complementary to scientific knowledge (UNISDR, 2015, p. 15). Despite criticism that this partial inclusion reduces TILK to its local and practical relevance shorn of its critiques of modernity and potential to produce an ‘epistemological’ shift away from the ‘certainties of Western science’ (Gaillard, 2019, p. S8), it does provide a meaningful starting point for engaging with communities and forms of knowledge that have been marginalised in discussions of ecological disaster. The focus on local and practical relevance sidesteps the issue of spiritual or metaphysical beliefs both within TILK and within dominant religious traditions, rendering the TILK framework ill suited to address Evangelical Christianity (EC). The unsuitability of including EC under the TILK umbrella is further illustrated by the role of Christianity in the advent of modernity and epistemic violence against traditional and local forms of knowledge.^2^

While TILK is not a suitable theoretical framework to consider the claims of EC, DRR advocates cannot simply ignore the challenge posed by the movement. Refusal to engage with spiritual traditions, both colonial and traditional/Indigenous, is not only practically counter‐productive, as evidenced by the success of EC's opposition to DRR, but it also reinforces what Lynch and Schwarz (2016, p. 636) refer to as the ‘secular fiction’ that frames responses to disasters and humanitarian emergencies ‘in secular terms only’, in which religion is viewed primarily in a negative manner. Furthermore, the TILK framework remains relevant to the discussion of EC because it demonstrates that treating different forms of knowledge only in a negative way ultimately hampers the success of DRR.

This paper addresses a pressing theoretical and practical question for DRR: how to deal with radical difference in perspectives on disaster in general, and EC perspectives, in particular. Drawing on a pluriversal viewpoint, I argue that it is possible to find ‘zones of contact’ that create possibilities for incorporating aspects of EC beliefs into the practice of DRR, while also providing a foundation to contest, oppose, and exclude fatalist, scapegoating, and conspiratorial disaster narratives. A pluriversal approach recognises radical difference without endorsing a relativist position, allowing for the articulation of basic standards for knowledge inclusion. In this case, I suggest that acceptance of pluriversality and a pragmatic commitment to ensuring stable and reliable natural processes on which human communities depend for their survival are basic standards for inclusion. Applying them to disaster claims within EC, this paper identifies points of connection and pragmatic engagement on humanitarian aid, creation care, and environmental stewardship, as well as areas of incompatibility pertaining to fatalist, scapegoating, and conspiratorial narratives. Importantly, I suggest that contestation of these narratives should be encouraged within EC and between DRR advocates and the EC community.

The paper focuses on EC for several reasons. Notably, it is a difficult test case for proponents of DRR and pluriversality, given Christianity's complicated relationship with modernity and its participation in epistemic violence against Indigenous knowledges. Nonetheless, EC is influential and spreading rapidly, particularly in the Global South, where it finds synergy with certain Indigenous cosmologies (Martin, 2017). EC's influence on disaster policies reflects its significant presence in global disaster relief as well as its increasing political mobilisation (Lynch, 2020), often in opposition to climate science and associated mitigation policies (Veldman et al., 2021). Notwithstanding this, EC is largely absent in the field of disaster studies. Scholars have examined disaster evangelism (Ensor, 2003; Lynch and Schwarz, 2016) and the impact of religious beliefs on risk perception, community preparedness, and resilience (Sims and Baumann, 1972; McGeehan and Baker, 2017; Hollis, 2020), but have paid far less attention to EC interpretations of disaster and their effect on political support for or opposition to DRR. Disaster studies still reflect a secular framework in which scientific and technical knowledge is dominant. By exploring EC perspectives on disaster, this study aims to demonstrate the terrain of contestation within EC, and to expand the number of cases examining alternative disaster‐related knowledge systems.

It is worth noting that my focus on EC precludes deeper engagement with a broader range of Christian perspectives on disaster, most notably the Catholic/liberationist tradition, which is more attentive to political ecology and Indigenous knowledge (Ensor, 2003; Martin, 2017; Lynch, 2020). It is beyond the scope of this paper to analyse this movement, although I would stress that this body of work demonstrates a more expansive range of possible interpretations of disasters, the environment, and Indigenous knowledge within the Christian tradition. These interpretations have yet to find resonance in EC owing in part to their association with Catholicism. Historically, EC has adopted an antagonistic position with respect to the Catholic Church, often rooted in conspiratorial, millenarian terms (Cohn, 1970; Patterson, 1988; Barkun, 2013).

The paper first examines the modernist/Christian foundations of the current international order to show how modernity displaced alternative knowledge systems, before turning to the centrality of knowledge production in the DRR framework, and the place of alternative knowledge systems within it. Next it explores knowledge claims about humanity/nature and the meanings of disaster in the EC community in North America, drawing on responses to Hurricanes Helene and Milton in 2024. It then investigates the pluriversal perspective that attempts to bring multiple knowledge systems into global governance in a manner that is neither hierarchical nor relativist. The paper concludes that disaster scholars and policymakers must look for and nurture pluriversality in alternative knowledge systems, find value in disaster analogies, and identify areas of practical engagement. This requires, at the very least, a rethinking of the modernist orientation to spiritual knowledge claims that dismisses them outright as premodern and backward.

## 2 MODERNITY AND KNOWLEDGE OF DISASTERS

The field of DRR has been described as a ‘battlefield of knowledge’ (Gaillard and Mercer, 2013. p. 93) in which different understandings of disaster compete to shape public policy and community behaviour. EC is an active participant in this field of contestation and offers an interpretation of disaster that stands in stark contrast to the dominant interpretation underpinning DRR. And yet, the foundations of DRR reflect a historical entanglement with an understanding of nature emergent from Christianity. Liberal modernity ushered in a key transformation through the disenchantment of nature and construction of a binary that placed humanity separate from, and dominant over, nature (Blaser, 2013; Hoffman and Oliver‐Smith, 2019). Liberal modernity's pursuit of human mastery over nature has its roots in Judeo‐Christian ‘dominion theology’ in which God granted humanity ‘free control over nature and its resources for their gain’ (Khamrang Varah et al., 2021, p. 85). Christian theology worked alongside secular science and philosophy to create the ‘cognitive monocentrism’ foundational to liberal modernity (Mignolo, 2018a, p. 92). The ‘discovery’ of Indigenous populations living in a purported ‘state of nature’ was a pivotal moment in transforming Christian dominion theology into political philosophy justifying colonisation and modernisation (Parasram, 2018). Christianity played a key role in the emergence and dominance of liberal modernity and co‐participated in epistemic violence against Indigenous knowledge (Lynch, 2020). Yet, as Lynch (2020, p. 18) shows, conflating Christianity, especially Evangelical variants, with liberal modernity is problematically reductionist and ignores significant philosophical and pragmatic antagonisms.

A core antagonism emerges from the liberal, modern ontology foundational to DRR that rejects the idea of ‘enchanted nature’ and insists on human agency as the central explanation for, and solution to, disaster. The disenchantment process depicted nature as a set of objective, mechanical laws discoverable through scientific analysis and controllable through technological progress and social reordering (Scott, 1998, p. 4; Berkes, 2012, p. 2). Disasters were no longer understood as a manifestation of divine intent but rather as the result of processes that humans could study, measure, predict, manipulate, and control through universally applicable solutions (Hewitt, 1983). In pursuit of these goals, modernity sought to reduce human vulnerability through the establishment of centralised political authority in the form of the secular state (Meyer et al., 1997), and epistemic authority in the form of specialised scientific knowledge. The success of scientific fields in producing technological innovations ‘contributed plausibility to the emergent story of modernity as a progressive movement marked by the mastery of nature’ and became the foundation of a ‘regime of knowledge that claimed to be able to get to the Truth while disqualifying all others as beliefs’ (Blaser, 2013, p. 555).

Knowledge systems that held an enchanted view of nature linked human activity, non‐human agency, and reliable access to natural resources, justifying systems of governance that placed limits on human exploitation of nature (Grandjean et al., 2008, p. 193). The advent of modernity rendered these enchanted knowledge systems and their systems of governance a barrier to human progress that would disappear as societies developed and progressed along the timeline of modernisation (Casanova, 2009; Grosfoguel, 2013a; Parasram, 2018). While these societies still produced technological innovations and engaged in human management of natural processes (Henry, 1997; Kölbl‐Ebert, 2009; Attar, 2010; Jacobsen, 2013), they mostly did so in a way that imposed limits on human extraction of value from nature. In contrast, modernity reflected dominion theology in treating nature as a limitless object of exploitation in service of human prosperity (Falk, 1972; Borrows, 2018; Turner and Spalding, 2018; Escobar, 2018, p. 73).

The colonial modernising project entailed both physical and epistemological violence (de Sousa Santos, 2015), as colonised peoples were ‘forbidden from thinking, praying or practicing their cosmologies, knowledges and world views’ (Grosfoguel, 2013b, p. 84). Christian philosophy and practice co‐produced this epistemic violence, yet Christian thought was also marginalised by this process, primarily through relegation to the private sphere (Keane, 2000; Calhoun, Juergensmeyer, and VanAntwerpen, 2011; Agensky, 2017). The Lisbon (Portugal) earthquake of 1755 is illustrative of this shift, as Enlightenment thinkers challenged Christian interpretations of disaster as punishment by God and asserted the authority of secular, scientific authorities over religious authorities (Paice, 2008; Shrady, 2008). Modernity replaced the Christian concept of salvation with a secular view of progress (von Wright, 1997, p. 5) made possible by scientific discovery and technological development (Du Pisani, 2006). Traditional knowledges, including various spiritual traditions, were deemed ‘irrational’, belonging to a ‘superseded stage’ of development (Casanova, 2009, p. 1052).

The scientific study of natural processes contributed to impressive technological and societal achievements, as well as novel insights into disasters. High modernism introduced the concept of ‘natural disasters’ as naturally occurring, indiscriminate events independent of social systems (Tierney, 2007, p. 506). This conceptualisation gave way to a view of disasters as the result of natural hazards that differentially affect groups within societies due to socially, economically, and politically produced vulnerabilities (Spector and Kitsuse, 1987; Dynes, 1993; Blaikie et al., 1994; Cutter, 1996). Although Christian progressive reform movements played a role in connecting modern economic activity with disasters and the development of the welfare state (van Kersbergen, 2003), the evolution of the concept of disaster elevated state authority over disaster response and prevention (Platt, 1999) and scholarly examination of social factors. The vulnerability approach drew attention to the risks generated by social and economic structures that exposed different groups within a society to various hazards (Cannon, 1994, p. 16). The dominant understanding of disaster shifted from naturally occurring events to an intrinsic feature of modern capitalism and historical processes of marginalisation (Hewitt, 1983; Pelling, 2003; McEntire, 2004; Tierney, 2007; Hewitt, 2009). While the full impact of this post‐modern shift is still ongoing, the DRR framework is the most significant international effort to wrestle with the role of modern capitalist structures in producing disaster risk and vulnerability (Varley, 1994; Wisner, 2003; Hannigan, 2012; Gaillard, 2021), and set as its first priority the production of scientific knowledge.

The SFDRR's first call to action globally is to ‘enhance the development and dissemination of science‐based methodologies and tools to record and share disaster losses and relevant disaggregated data and statistics, as well as to strengthen disaster risk modelling, assessment, mapping, monitoring and multi‐hazard early warning systems’ (UNISDR, 2015, p. 16). All nine strategies at the global level address scientific and technical solutions and data production, while at the national level, the SFDRR calls for ‘dialogue and cooperation among scientific and technological communities, other relevant stakeholders and policymakers to facilitate a science‐policy interface’ (UNISDR, 2015, p. 15). The framework prioritises scientific knowledge production and promotes the expansion of this across and within societies.

Producing universalisable knowledge in global governance frameworks necessitates clarification of the relationship between different forms of knowledge found in the international realm, often in hierarchical terms (Muppidi, 2004, p. 274). DRR accomplishes this through the articulation of a science/non‐science ternary comprised of: (i) dominant scientific knowledge; (ii) local/practical TILK included when complementary (Kelman, Mercer, and Gaillard, 2012; Tozier de la Poterie and Baudoin, 2015; Oliver‐Smith, 2016; Hadlos, Opdyke, and Hadigheh, 2022); and (iii) other forms of knowledge excluded as irrelevant, outmoded, and superstitious (Blaser, 2013; Balay‐As, Marlowe, and Gaillard, 2018). The hierarchical ranking of knowledge risks reinforcing a modernist perspective that places societies and subgroups within societies on a historical timeline in terms of their trajectory towards full modernity (Muppidi, 2004, p. 274; Blaser, 2013, p. 549), and turning ‘the “disorderly,” the “failed” and the “poor” into the *objects of governance* of the “orderly,” the successful, and the rich’ (Muppidi, 2004, p. 276). Consequently, scholars have begun to examine critically the science–TILK distinction.

As noted earlier, EC does not fit comfortably into the TILK framework, but the existing literature exploring the science–TILK distinction in DRR is helpful with respect to analysis of EC because it challenges the idea that only scientific knowledge is changing/adaptable, prioritises assessment of underlying values, and draws attention to questions of governance and state authority. The science–TILK hierarchy problematically presents scientific/technological knowledge as constantly improving and evolving in response to new discoveries while presenting TILK as inter‐generational and unchanging, denying its dynamism, ‘continuous adaptation’, and hybridity (Hilhorst et al., 2015, p. 510; Hadlos, Opdyke, and Hadigheh, 2022). Defining a knowledge system as unchanging risks rendering it less relevant in the context of rapid change. Conversely, recognising that all knowledge systems change over time and adapt to new settings and information encourages analysis of how EC has altered over time and potentially how it could further change in ways supportive of DRR.

In addition, the science–TILK ternary conditions inclusion of TILK based on local, practical relevance, excluding underlying values and priorities at odds with liberal modernity (Kelman et al., 2012; Hadlos, Opdyke, and Hadigheh, 2022, p. 509). Inclusion conditional on practical relevance allows policymakers to extract ‘valuable fragments of knowledge’ divorced from larger knowledge and value systems (Hilhorst et al., 2015, p. 509). As Blaser (2013, p. 556) notes, restricting discussion to the realm of policy prescriptions based on a narrow set of issues excludes broader political and socio‐economic change. The scientific/technocratic language that precludes discussion of values is itself value‐laden. It reinforces the values and practices of modern neoliberalism (Blewitt, 2008; Reid, 2012; Alexander, 2013; Joseph, 2013; Schmidt, 2014; Carrigan, 2015, p. 133; Tierney, 2015; Adelman, 2018) that prioritise economic growth, returns on investment, profitability, and long‐term risk calculations over humanitarian concerns or the strengthening of local communities (Hannigan, 2012; Hollis, 2020). Placing greater attention on values, and the dominance of neoliberal principles in particular, encourages further exploration of how neoliberal thinkers draw on a concept of ‘Christian civilisation’ to justify the neoliberal project (Slobodian, 2025) and how EC has increasingly embraced neoliberal capitalism over other values consistent with Christianity (Alberta, 2023).

Critical examination of the science–TILK hierarchy also highlights the complicated matter of governmental authority. As Starblanket and Stark (2018, p. 179) note, the partial inclusion of Indigenous knowledge in ‘state‐driven projects’ risks co‐optation of this knowledge by governmental actors that have violently marginalised Indigenous peoples and knowledge. While EC has not experienced the marginalisation of Indigenous communities, the EC community is increasingly mobilised through the perception of marginalisation and exclusion at the hands of the secular state (Alberta, 2023). Many aspects of DRR strongly enhance the authority and reach of the state. The second priority of the SFDRR calls for ‘strengthening disaster risk governance’ at ‘the national, regional and global levels’ for ‘prevention, mitigation, preparedness, response, recovery and rehabilitation’ (UNISDR, 2015, p. 17). DRR includes initiatives on health, education, poverty alleviation, gender, age, and ability, and incorporates the private sector and civil society as relevant stakeholders alongside governments. It targets multiple levels of governance, ranging from the local, regional, transboundary, state, and global (UNISDR, 2015) and incorporates procedural inclusiveness alongside paying greater attention to health, mobility, development, and climate change (Aitsi‐Selmi et al., 2015; Guadagno, 2016). DRR calls for potentially massive governmental intervention to address these structural issues (see, for example, Hewitt, 1983; Blaikie et al., 1994; Pelling, 2003; Oliver‐Smith, 2016; Kelman, 2020; Gaillard, 2021).

DRR expands the scope of governmental authority, and brings civil society actors, including faith‐based organisations (FBOs), into the global DRR framework and governance structure. The expansion of global governance has generated an intense backlash against the associated intrusion of governmental authority into increasingly more areas of public life (Börzel and Zürn, 2021; Walter, 2021). EC has a long history of opposing the expansion of secular (state and scientific) authority wherever it manifests: locally, nationally, and globally (Worthen, 2014; Fitzgerald, 2018). It shares with a growing number of libertarian and populist movements around the world concerns about the expansion of global governance and state authority (Löfflmann, 2022; Wajner, 2023), leading to strong levels of support among the EC movement for populist leaders (Yilmaz, Morieson, and Demir, 2021; Pally, 2022). The DRR framework presents global governance and enhanced state capacity as an uncontestable positive, whereas EC has aligned with populists in interpreting these developments negatively.

The DRR framework, as with most international governance frameworks, is mostly silent on the issue of religious belief. The SFDRR includes a single clause acknowledging the importance of protecting religious sites to foster community resilience (UNISDR, 2015, p. 19). Existing scholarly work has been much more attentive to how spiritual traditions shape disaster risk perceptions, readiness, and response; even these studies, however, have largely overlooked radically different understandings of disaster. Numerous scholars have noted that certain religious perspectives contribute to a fatalistic attitude that hinders disaster preparation (Sims and Baumann, 1972; Chester, 1998; Chester and Duncan, 2010; Gaillard and Texier, 2010), while others demonstrate the positive influence of religious belief in facilitating personal and communal recovery post disaster (Fountain, Kindon, and Murray, 2004; Massey and Sutton, 2007; Schipper, 2010; Oliver‐Smith, 2016). Additional studies show that religious beliefs interact with other belief systems and structural factors to mould disaster risk assessment and response. McGeehan and Baker (2017) underline that there is no singular religious/spiritual relationship with DRR, while Hollis (2020) reveals significant variation between, and within, religious traditions in how they understand and impact disaster. Gaillard and Texier (2010) show that religious beliefs about disaster reflect the local cultural, political, and economic context. In response, these authors argue that DRR practitioners need to understand better these local settings and how religious beliefs interact with other local contributors to vulnerability. While I agree that increased understanding of religious interpretations of disaster is an essential first step towards identifying potential points of overlap and tension, comprehension often highlights irreconcilable difference. Moreover, religious beliefs are not just local; the EC community is nationally organised in the US, and has a mounting presence globally. It is insufficient for DRR practitioners to focus exclusively on local manifestations of religious communities' responses to disasters when these are profoundly shaped by a national, or even transnational, movement.

## 3 EC AND DISASTERS

EC contains a variety of understandings of disaster, reflecting its highly decentralised character and resistance to a single overarching epistemic authority (O'Mathúna, 2018). It emphasises individual salvation rooted in personal experience and a lay interpretation of scripture, with a deep‐rooted suspicion of centralised authority, secular or religious (Fitzgerald, 2018). This has resulted in a plethora of EC denominations, churches, and splinter groups that differ substantially on theological matters and political issues. These differences have produced repeated schisms and intense polarisation regarding some of the defining political questions in the US. Evangelicals have taken opposing views on slavery, women's suffrage, worker's rights, civil rights, desegregation, the welfare state, abortion, social justice, and the War on Terror (Fitzgerald, 2018), as well as, more recently, the return of President Donald Trump to the White House. To be sure, at certain historical junctures, a majority within the EC community have adopted common positions: over the past three decades, EC has cohered on matters of reproductive rights, sexual politics, and the authority of the state generally (Fitzgerald, 2018), but even on these questions, there are disagreements within the movement.

One of the key axes of division within EC concerns modernity. The anti‐modernist strand is rooted in supernaturalism and divine intervention, a literal (inerrant) reading of the Bible, a rejection of science, and a belief in the ultimate and imminent demise of human civilisation—the end times. The modernist component, in contrast, endorses scientific research as an illumination of God's methods, a figurative reading of the Bible grounded in modern historiography and hermeneutics, a rejection of supernaturalism, and a progressive view of human civilisation (Fitzgerald, 2018, p. 72). Many deliberations within EC reflect this overarching division; yet, debates between modernist and anti‐modernist EC elements are mutually intelligible and hold potential for establishing common ground because they have a shared foundation based on a metaphysical conception of time (Hollis, 2020; James and Steger, 2023), a ‘two kingdom’ ontology, and a view of nature as ‘fallen creation’. The evangelical metaphysical conception of time constructs human history in terms of Jesus' arrival on Earth and the subsequent progression towards the ‘end times’ and his return. There are substantial disagreements within EC about literal or metaphorical interpretations, the order in which specific events are supposed to occur (Pre‐Millennialism, Post‐Millennialism, or Amillennialism), and the current point in time in this historical process (James and Steger, 2023). This metaphysical conception of time rests on a ‘two kingdom’ ontology in which reality consists of two interconnected worlds: the earthly and the spiritual. The earthly world functions largely in accordance with a modernist ontology and time frame, as well as scientific explanations, whereas the spiritual world involves the continuing battle between the forces of good and evil that takes place outside of, but interacts with, the material world. Consequently, events witnessed in the earthly realm may be explained historically/materially and/or spiritually.

The EC ontology understands nature as ‘fallen creation’. The Judeo‐Christian creation narrative (as captured in the Book of Genesis) describes the universe, Earth, and all life on the planet as brought into being by an omnipotent deity^3^ over a six‐day period that culminated with the creation of humanity. Humanity is part of creation but holds a unique and special place within it, giving rise to differing interpretations of its relationship with and moral obligation towards creation. One interpretation depicts humans as stewards of creation (Eom and Ng, 2023), while a second depicts humans as having dominion over it (Khamrang Varah et al., 2021). The stewardship model imposes a duty on humans to take care of God's creation, whereas the dominion model asserts that God gave humanity ‘free control over nature and its resources for their gain’ (Khamrang Varah et al., 2021, p. 85). As noted earlier, the dominion view of nature within Christian thought has profoundly shaped the modernist project. More recently, however, theologians and environmental activists have begun to stress the stewardship model as a point of connection between various religious traditions, including Christianity, and sustainable development/ecological justice (Eom and Ng, 2023; McLeod et al., 2024).

In addition to depicting nature as creation, EC understands creation as ‘fallen’. The ‘Fall’ refers to the moment of original sin committed by Adam and Eve in the Garden of Eden, and attempts to resolve the problem of suffering in a world created by a perfect and loving creator. While Christian theologians have spent a great deal of time on the problem of suffering, the ‘Fall’ narrative presents nature as no longer operating as the creator initially intended due to humanity's original sin (Steinberg, 2006; White, 2016, O'Mathúna, 2018). Humanity is ultimately responsible for the suffering caused by both natural processes and human action, rather than the creator. Disasters demonstrate that the Earth is ‘fallen’ and EC contends that it will be replaced by a new Heaven and Earth in which there will be no pain, suffering, or ‘natural disasters’ (Steinberg, 2006; White, 2016; O'Mathúna, 2018; Walker, 2024). In this worldview, disasters are still seen as indiscriminate ‘natural disasters’, but with the view that the fallen Earth cannot be saved, and disaster and suffering cannot be eliminated. Individuals can be saved through their Christian faith, but this does not mean that they will be spared suffering. Consequently, the arc of human history moves towards the final redemption of both humanity and creation, which in some strands of EC involves the literal destruction and replacement of the Earth: a fatalist perspective openly hostile to risk reduction or environmental sustainability.

This larger worldview shapes how adherents produce meaning in response to specific disasters, with a number of recurrent explanations in EC ranging from disasters as punishment to disasters as producing a greater good (O'Mathúna, 2018). In my analysis of the EC response to Hurricanes Helene and Milton,^4^ I identity three disaster explanations/analogies: punishment; theodicy; and conspiracy. Historically, a common disaster analogy in EC (as well as in other religious traditions) presents disaster as *punishment* by God (Shimoyama, 2002; Udías, 2009; Cox et al., 2018). In the EC tradition, several biblical stories (such as the Flood and Sodom and Gomorrah) imply that God uses disaster to punish humanity or specific communities for their sins. The punishment narrative can be used to scapegoat particular groups as responsible for God's wrath, and to promote moral purification of the community. A prominent recent example of this in the US involved Pat Robertson, a popular televangelist who founded the Christian Broadcasting Network and unsuccessfully ran for the Republican nomination for president in 1988. Robertson regularly described disasters as divine punishment. He claimed that California might be struck by disasters for Disney hosting LGBTQ+ (lesbian, gay, bisexual, transgender, queer/questioning, plus other identities) days (Bentz, 2013), that Hurricane Katrina in 2005 was punishment for America's permissive policies on abortion, and the Haitian earthquake in 2010 was the result of a ‘pact with the devil’ the Haitians had struck in their decolonial struggle for freedom from the French (Zajac, 2010). Televangelist John Hagee depicted Katrina as punishment for US policies on abortion and homosexuality; in 2012, furthermore, several EC leaders in the US claimed that Hurricane Sandy was punishment for the legalisation of gay marriage (M.R., 2012). Multiple EC pastors attributed the destruction caused by Katrina to immorality in either the city of New Orleans, Louisiana, or the US more generally (Walker, 2015).

In more recent disasters, the scapegoating narrative appears less common among prominent evangelical leaders. Although they still appear on social media, in contrast to the Pat Robertson years, no prominent evangelical publication or leader has endorsed this position publicly in response to disasters. For instance, the National Association of Evangelicals released a video on Hurricanes Helene and Milton that does not mention the punishment narrative, focusing instead on humanitarian relief and the importance of strengthening local churches. A small number of EC leaders did try to dispel punishment claims. After Helene and Milton caused significant damage in Florida and North and South Carolina in 2024, social media posts blamed the destruction of Asheville, North Carolina, on its liberal perspectives on sexuality (Wolfe, 2024). Russell Moore, writing in *Christianity Today*—regarded as ‘the closest thing in evangelicalism as a journal of record’ (Gribben, 2021, p. 72)—published an article to repudiate these claims (Moore, 2024). Even fundamentalist evangelicals downplayed this interpretation, even if they did not fully repudiate it. William Wolfe (2024), who served in the first Trump administration (2017–21) and writes for the Standing for Freedom Center (based at Liberty University in Lynchburg, Virginia, and connected with the highly influential Falwell family), and who has been highly critical of Moore (Alberta, 2023), cautioned against attributing destruction in any particular community to its sins. While Wolfe and Moore offered different viewpoints on the legitimacy of the punishment analogy, both advocated a humanitarian response rather than scapegoating.

The humanitarian response to disaster is the dominant perspective within EC, and reflects the long tradition of *theodicy* that reconciles belief in a perfect and all‐powerful God with the existence of human suffering and disasters. There are two basic strands within theodicy that explain suffering: (i) God has given humans free will and the ability to make choices that can result in suffering; and (ii) God allows disasters to happen to achieve a greater good (Chester, 1998, 2005; O'Mathúna, 2018). In the case of disasters triggered by natural hazards, theodicy suggests that God allows such events to happen for some greater good that may not be apparent to humanity (Kaell, 2022). Theodicy rejects the punishment narrative and positions disaster and suffering as inevitable features of the human condition in a fallen world that affects everyone indiscriminately. It suggests that disasters can lead to the 'greater good’; interpreted in EC as reinforcing and spreading Christianity, either in the form of disaster evangelism or renewed personal faith and practice.

In response to Hurricanes Helene and Milton, Moore (2024) emphasised the ambiguity and mystery of ‘natural evil’ in the world, noting that disasters affect all communities and that assigning blame for them is inconsistent with the Christian worldview. He calls for a pragmatic response based on living the Christian faith: clearing away fallen trees, supporting those who are grieving, and providing food and water to those who need it (Moore, 2024). Similarly, Wolfe (2024) underscores three evangelical perspectives on disaster: (i) the fallen nature of creation; (ii) non‐discrimination between Christians and non‐Christians; and (iii) the importance of personal repentance. As with Moore, Wolfe (2024) acknowledges the ambiguity of Christian beliefs about disaster, in that they affect the ‘just and unjust’, and calls for pragmatic measures: pray for those impacted, support humanitarian relief, and volunteer to help those affected.

While the responses to Helene and Milton demonstrated the importance of theodicy and humanitarianism domestically, this perspective is even more firmly rooted in the international realm. EC promotes participation in foreign missions as a way of enacting one's Christian faith, helping fellow Christians in distress (Kaell, 2022), and as an opportunity to evangelise (Chester, 1998, p. 490). Since the early twentieth century, humanitarian relief has been the cornerstone of the evangelical humanitarian network (Barnett and Stein, 2012). For Christian organisations, humanitarian aid became a central pillar of their outreach, providing an opportunity to practise one's faith and to engage in disaster evangelism (Ensor, 2003). For many EC humanitarian groups, the separation of the political from the religious, the suspicion of state power, and support for neoliberal capitalism encouraged commitment to the alleviation of human suffering rather than addressing structural factors that produce vulnerability (Barnett and Stein, 2012; Rozario, 2019). There are ongoing debates within EC about the morality of proselytising or providing conditional aid following disasters, but EC groups consistently portray their participation in international disaster relief as a chance to promote or defend Christianity (Ensor, 2003; Lynch and Schwarz, 2016). EC FBOs have hesitated to restrict their mission based on principles established by secular authorities, and, tending towards the conspiratorial, interpret the increasing prohibition on disaster evangelism as part of a larger trend of opposition to Christianity (Lynch and Schwarz, 2016).

*Conspiracism* is a third disaster narrative common within EC, and one that appears to be growing. The literature on EC (and religion, more broadly) and on DRR largely neglects the problem of conspiracism (O'Mahuna, 2018). It presents preparations for, and responses to, disaster as part of a plot orchestrated by a secret group working on behalf of the forces of evil. Although evangelicals are not the only group to embrace conspiracy theories, there are aspects of the EC worldview founded on conspiratorial thinking, especially its millenarian strands that view time progressing towards the Apocalypse, with world events orchestrated by the anti‐Christ (Barkun, 2013; Wood and Douglas, 2018; van Eck Duymaer van Twist and Newcombe, 2018; MacMillen and Rush, 2022). In fact, disasters and social breakdown are so foundational to EC that Barkun (2011, p. 29) characterises the community as having a ‘disaster obsession’. In the conspiratorial narrative, disasters are either a sign of the end times or an event that precipitates the forces of evil consolidating control over society. This connection, Barkun (2013) notes, has often placed the Federal Emergency Management Agency (FEMA) at the centre of EC conspiratorial claims in the US, and in some instances encourages a politics of control to secure Christian control of the government.

These aspects of conspiratorial thinking were evident in the aftermath of Hurricanes Helene and Milton. In Wolfe's (2024) assessment of these events, he identified two popular interpretations: (i) climate change; and (ii) punishment of the city of Asheville for its liberal sexual politics. He rejects both explanations but cautions that they are ‘not equally offensive or equally wrong’, asserting that ‘“climate change” or “global warming” isn't real. It's a fabricated crisis that is weaponized by the global elites’. Alternatively, he claims that God has used disaster to punish communities in the past for sexual immorality, so the punishment narrative is more plausible than the climate change narrative. Wolfe's fundamentalist strand of EC explicitly rejects the science of climate change as a conspiracy by ‘global elites’ and endorses a political movement that eschews worldwide coordination and climate science, in favour of gaining control of the state to undermine or eliminate governmental disaster response capacity. Suspicion of climate science is not a marginal position within EC, as it has gradually shifted against environmentalism and become more strongly identified with the political right and the Republican Party (Pogue, 2024). Evangelicals in the US are the least likely group to believe in climate change or to support climate action, a position that derives in part from their belief in the inevitable destruction of the Earth (Veldman et al., 2021). In recent years, this climate scepticism has combined with conspiratorial aspects of EC to support a variety of contemporary claims, such as those of QAnon (Beauchamp, 2022). Several evangelical actors have promoted conspiracy theories and mobilised around Trump's presidential candidacy and a return (or establishment) of an explicitly Christian state in the US (Berry, 2020).

These groups reject climate change and have been at the forefront of assertions regarding Hurricanes Helene and Milton (Butler, 2024), notably presenting the disasters as the product of governmental manipulation of the weather and/or as the intentional failure of FEMA to provide assistance (Butler, 2024). FEMA reported that the present level of misinformation is the worst it has ever experienced and has had to issue multiple statements to dispel misinformation (FEMA, 2024; see Feldman and Ohnsman, 2024). These conspiracies resulted in a backlash and death threats against meteorologists in the US, as well as calls to destroy meteorological equipment (Millman, 2024). A conference to improve emergency response to future disasters was cancelled owing to harassment by conspiracists who asserted that the group organising the conference was actively planning the next disaster (Thompson, 2024). While conspiratorial claims that the government causes hurricanes are marginal, conspiracies that undermine trust in FEMA and weather/meteorological organisations are spreading, and are connected to the widespread scepticism of climate science and the centrality of conspiracism among some evangelical groups.

EC conspiracism and distrust of ‘mainstream’ institutions has also influenced the Christian reconstructionist movement, which seeks to reconstitute regions of the US as distinct political communities founded on an originalist reading of the Constitution, severely limited government, and millenarian Christian theology (Gribben, 2021). Reconstructionism emphasises physical separation from modern society through migration to remote/rural areas to prepare for and survive impending disasters and societal collapse (Gribben, 2021). As one might imagine, disasters are central to this narrative, signalling the larger societal collapse associated with the reign of the anti‐Christ. For reconstructionists, the appropriate response to disaster is to draw on preparations and engage in self‐sufficiency; this is an extremist version of disaster resilience in which the evangelical community's role is not to prevent collapse or assist with rebuilding, but to survive. This worldview is highly local and pragmatic in its orientation, and rejects engagement with modern, secular society. Reconstructionists see modern society as the source of vulnerability and disaster; rather than live in a modern society or reform/improve it, they advocate for separation and the development of local capacity. The creation of jurisdictional separation can produce difficult engagements between these communities and their non‐reconstructionist neighbours (Gribben, 2021), but also resilience among them; it does not insist on dominating or controlling others.

The diversity of perspectives on disasters among evangelicals, and the radical difference compared to modernist understandings, highlights the challenges that DRR advocates face in promoting disaster governance and risk reduction at a national and global scale. Aspects of EC ontology and narratives of disaster are not complementary to scientific knowledge of disasters, and tend towards scapegoating, fatalism, or conspiracism. The liberal modern perspective rejects these accounts and insists on the superiority of liberal modernity, largely refusing to engage with beliefs considered superstitious and backward. In short, it reinforces the modernist hierarchy of knowledge. Instead of continuing to enforce the dominance of liberal modern perspectives, I argue that a pluriversal approach that seeks ‘zones of contact’ makes pragmatic engagement on key aspects of DRR possible, while facilitating a standard for assessing the contribution (and therefore inclusion) of alternative knowledge claims.

## 4 PLURIVERSALISM

Emerging from post‐colonial scholarship, pluriversality asserts that reality is composed of multiple interconnected worlds (or processes of worlding) (Mignolo, 2018a). From this perspective, the assertion that reality consists of one unified world made knowable exclusively through scientific knowledge is part of the colonial project that destroys alternative ways of producing knowledge (Chakrabarty, 2007; Grosfoguel, 2013a; de Sousa Santos, 2015). Pluriversality accepts that reality cannot be captured by any single perspective; people ‘access the same reality from different places and thus either see, or experience a different slot of the same reality or … a different reality altogether’ (Reiter, 2018, p. 6). William James (1977, p. 167) observed that pluriversality draws attention to the interconnection between worlds, underlining that ‘things are with one another in many ways, but nothing includes everything or dominates over everything’.

Consequently, pluriversality rejects the dominance of one worldism and supports the ‘coexistence of many worlds’ that are necessarily entangled (Oslender, 2018, p. 138). Acceptance of multiple worlds is ‘a substantive value in itself’ and opposes the liberal modern project of creating a single shared understanding of reality (Hutchings, 2019, p. 119). While there will be some shared experiences of reality at key points of interconnection—what Escobar (2018, p. 83) refers to as ‘zones of contact and partial common ground’—these are always partial, and not all experiences are mutually comprehensible. Thus, adopting a pluriversal perspective requires abandoning the liberal, modern view that all of humanity fundamentally shares the same world, that we can fully understand each other, and that we are destined to become liberal and modern. The goal is to create zones of contact and mutual intelligibility on a pragmatic basis. Attempts to impose unity or a single view of reality does not do so, but instead ‘leads to resistance, further repression and disunity’ (Tully, 1995, p. 197). Pluriversality requires a level of comfort with radical difference and ambiguity, whereby people ‘keep a foot in two or more worlds, straddling perspectives to maintain tension between them’ (Connolly, 2005, p. 4).

The issue of prayer is an illustrative example. Many Christians and adherents of other faith groups offer prayer before, during, and after disasters, and view this as an active response than can produce results (Mitchell, 2003; Kaell, 2022). From a modern, secular perspective, offers of prayer may be regarded benignly as thoughtful and well‐meaning but without effect, or more disparagingly as superstitious, ignorant, and a poor substitute for structural change (Thunström and Noy, 2019). Alternatively, a scientific approach may test prayer's measurable effects on individual well‐being and/or society's sense of community, resilience, disaster preparedness, and capacity for recovery (see, for example, Ladd and Spilka, 2002; Njus and Scharmer, 2020). The benign and dismissive perceptions of prayer and some scientific studies reinforce a ‘one world’ approach that insists on defining the practice exclusively on its terms, while denying the knowledge claims of those participating in the activity. A pluriversal perspective accepts that those who hold a secular modern worldview cannot fully understand prayer and therefore cannot define it exclusively on their own terms. Importantly, the scientific study of prayer can be consistent with a pluriversal approach. Scientists create a zone of contact by taking seriously the claim that prayer has meaningful impacts and that some of these are accessible through scientific conventions for knowledge production, while acknowledging that the full effect/experience of prayer exceeds the parameters of scientific study (Spilka and Ladd, 2012).

Two challenges that emerge from a pluriversal perspective are how to address claims to universality and how to distinguish between different knowledge claims. Some versions of pluriversality suggest that worlds that insist on universality are incompatible with others (Hutchings, 2019, p. 118; Schaarsberg, 2024). In contrast, Mignolo (2018b, p. x) argues that ‘all known civilizations have been founded on the universality of their cosmologies’, and thus we cannot exclude knowledge systems based on their claims to universality. He adds that pluriversality is itself ‘a universal project’ that rejects the idea that the only way to understand and act in the world is by asserting a ‘unified totality’ (Mignolo, 2018b, p. x). The problem with liberal modernity is not its claim to universality, but the insistence that its universality be recognised as superior (Mignolo, 2018). For Mignolo (2018b, p. x), ‘Western universalism has the right to coexist in the pluriverse of meaning’, but as one of many cosmologies, and not to dominate or exterminate others. It requires a recognition of the limits of one's own system of knowledge, genuine dialogue with other systems of knowledge, and a willingness to accept and embrace ambiguity.

At the same time, pluriversalism is not a relativist position that accepts all worldviews and epistemologies as equally valid (James and Steger, 2023). Knowledge systems are essential to provide meaning, but not all knowledge systems are equally valid or as good at explaining the world (Reiter, 2018, p. 2). James (1977, p. 164) conceded that there are ‘inferior books, bad statues, dull speeches, [and] tenth rate men and women’, and that in assessing knowledge, ‘there must be extrication, there must be competition for survival, but the clay matrix and noble gem must first come into being unsifted’. In short, the value of a worldview and its inclusion in the pluriverse must rest on an assessment of its validity or usefulness. Harding (2008, p. 6) similarly stresses the need for ‘realistic reassessments’ of all knowledge systems rather than ‘romantic evaluations or demonizations’. Blaser (2013, p. 552) suggests that some ontologies can be ‘wrong’, not in the sense of the accuracy of their depiction of ‘ultimate reality’, but by ‘enacting worlds in which or with which we do not want to live’.

With respect to DRR, I suggest that there are two essential starting points for inclusion: (i) recognition that human societies depend on stable and reliable natural processes associated with healthy ecosystems; and (ii) that no single nature/human analogy or narrative of disaster is dominant or sufficient on its own. The recognition that the survival of human communities depends on stable and predictable access to natural resources is common to liberal modernity and most, if not all, of the non‐modern societies it has sought to displace. Disaster narratives in these societies have highlighted human dependence on nature and sought to establish causal connections that would reduce human vulnerability to the vicissitudes of nature (Grandjean et al., 2008). Harding (2018, p. 44) similarly shows that the analogies societies use to understand the nature–human relationship all highlight the centrality of healthy ecosystems, even as they draw attention to ‘different features’ of the natural environment and the ‘appropriate ways to interact with’ nature. This recognition is evident in EC as well. Numerous biblical stories on disaster (floods, drought, famine, disease, and earthquakes) demonstrate the centrality of healthy, functioning ecosystems for the community's survival. As noted earlier, however, there are strands of EC that welcome the demise of the Earth and human societies; I argue that these worldviews do not share the basic common concern for the survival of human communities, are not consistent with pluriversality, and ought to be rejected and contested. Pluriversality requires accepting that some people believe the world is about to end and should not hinder their efforts to prepare for it, but it does not require accepting their active efforts to bring about social disorder. Pluriversality rejects perspectives that enact worlds ‘in which we would not want to live’ (Blaser, 2013, p. 552) and EC perspectives that enact or bring into being a world convulsed by the end times fall far short of this standard.

The second criteria emergent from the pluriversal perspective is that no single narrative/analogy is dominant. In many ways, EC recognises this through the dominion and stewardship analogies of the human–nature relationship. The concern with EC (and liberal modernity) is not the existence of the dominion analogy, but its exclusion of, or dominance over, the stewardship analogy. Pluriversality does not identify the only or the best narrative that captures the human–nature relationship, but sees value in each of the cultural analogies/narratives (Harding, 2018). The goal is not to replace the dominion model with another dominant analogy, but to place it alongside others, such as the stewardship analogy that promotes creation care. The dominion narrative may be helpful in emphasising the unique capacity of humans to extract value from nature and to modify the natural environment for their benefit, including reducing vulnerability to disaster risk. Alternatively, the stewardship model highlights the moral impetus of humanity to care for a creation they were gifted and tasked with preserving.

Within EC, there is a growing movement to promote the stewardship model. The most prominent organisation, the National Association of Evangelicals, acknowledges climate change and promotes the concept of ‘creation care’ as an evangelical response to the problem. Its *Loving the Least of These* report clearly lays out a Christian case to prevent the degradation of ‘God's creation’ and to ‘understand what causes natural disasters to be so terrible’ (Boorse, 2022, p. 23). This use of the stewardship analogy to care for creation provides common ground for pragmatic engagement on DRR. There are several evangelical groups, such as the Young Evangelicals for Climate Action, the Evangelical Environmental Network, World Relief, and A Rocha, that embrace an evangelical worldview as well as the legitimacy of climate science. They demonstrate engagement with pluriversality through their acceptance of scientific knowledge alongside evangelical knowledge claims, and endorse pragmatic action on climate change and environmental destruction. Support for concerted action on climate change is particularly appealing to younger generations of evangelicals (Leiserowitz, 2022; Lowe et al., 2022).

In foregoing efforts to produce or impose a shared reality, pluriversalism endorses a ‘hard‐nosed pragmatism’ that reveals interconnections between different worlds and temporarily creates a sense of unity (Blaser, 2013, p. 553; Savransky, 2021, p. 8). Disaster relief is a prime example of a pragmatic zone of contact between EC and liberal, modern worldviews. This zone of contact still contains tensions and differences over foundational principles, as well as the appropriateness of evangelism and aid conditionality (Lynch and Schwarz, 2016). Nevertheless, various organisations continue to cooperate in the realm of disaster relief, and many EC FBOs have taken a leading role in contesting proselytisation and conditionality in aid and in establishing internationally applicable standards that prevent some of the negative impacts of disaster aid.

Pragmatic concerns have also led evangelical leaders to contest scapegoating narratives resonant in EC (and elsewhere). The Moore and Wolfe interventions both emphasised the pragmatic benefits of disaster relief over the punishment narrative for enacting one's faith. Privileging the zones of contact enacted by theodicy and humanitarianism as well as by stewardship and creation care narratives may be the most effective way of connecting EC with DRR. I contend, however, that a pluriversal perspective may even recognise value in the punishment analogy by emphasising the moral dimensions of disaster. To be clear, the scapegoating of marginalised groups within society, as exemplified by Pat Robertson, is completely at odds with pluriversality, but the general idea that some human actions and actors hold some blame for contributing to disaster and suffering is not. This perspective can provide a corrective to the humanitarian response that prioritises the alleviation of suffering to the exclusion of addressing causal factors. There is a similar tendency in the climate change discussion to obfuscate the moral dimensions of disaster, by placing blame on a generic group, such as ‘the West’, ‘capitalists’, or ‘greedy corporations’ (Rudiak‐Gould, 2014), or by assigning responsibility to ‘climate change’ generically rather than specific local development or governance failures. While great care needs to be taken in assigning blame for weather‐related disasters, given their causal complexity (Hulme, 2014), the punishment analogy reminds society to consider the question of blame and responsibility for disaster, which may, in turn, lead to a greater focus on choices to create or maintain structural factors that contribute to disaster risk, which fits the theodicy tradition in EC.

Lastly, a pluriversal perspective may even identify value in the conspiratorial narrative common in EC. While specific EC conspiracies concerning the end times and the anti‐Christ are inconsistent with pluriversality, the conspiracy analogy draws attention to severe inequality in the distribution of wealth, power, knowledge, and authority, as well as the efforts by powerful actors to deceive for their benefit (Basham, 2019). Inequality and deception by powerful actors are so commonplace that conspiracy theories function as a zone of contact between radically different societies, worldviews, and religions, which is why they are evident around the globe and across the political spectrum. At their core, conspiracy theories act as a corrective to naive faith in the claims of powerful actors (Coady, 2019). They are a form of stigmatised knowledge that is deeply critical of dominant explanations (Barkun, 2015; Coady, 2019). This scepticism of dominant accounts, Uscinski (2018, p. 238) argues, plays an important role in democracies, acting as ‘defence lawyers’ that force the establishment to explain and defend its view. As with defence lawyers, Uscinski (2018, p. 238) adds, ‘they lose more often than they win’, but the value of opposing council is not in revealing truth (although they do on occasion) but in ‘incentivizing good behaviour by the powerful’ who know they are being ‘watched, investigated and publicized’.

To be clear, there is a large body of work highlighting the dangers posed by conspiracy theories. As an ontology of ‘repressive suspicion’, conspiracy theories foment suspicion of dominant powers while producing or reinforcing oppressive structures and limiting human freedom (Conway, 2025). This is evident in the EC community's commitment to seemingly uncritical support of neoliberal capitalism. The growth of conspiracy theories should raise concerns that segments of society are becoming supportive of greater oppression and systems that produce inequality and vulnerability; however, they also demonstrate that key institutions of governance are losing legitimacy and doing an insufficient job of defending their worldview and institutions. As certain actors profit from disasters and post‐disaster policies (Klein, 2007; Loewenstein, 2015), healthy scepticism of disaster policies is warranted; and the rise of unhealthy scepticism in the form of conspiracy theories should ring an alarm bell’ in democracies (Uscinski, 2018, p. 238).

## 5 CONCLUSION

The deliberate dismantling of DRR in the US is currently underway, facilitated and supported by the EC community. The Trump administration has implemented significant cuts in relation to FEMA, the National Weather Service, and climate research across a range of federally funded bodies (Moreno, 2025). FEMA (2025) has eliminated its Building Resilient Infrastructure and Communities programme to place greater emphasis on disaster relief programmes. These changes undermine DRR in the US and will likely have a global impact. It is not coincidental that the Trump administration has also given prominent roles to evangelical nationalists and reconstructionists, as key architects of Project 2025, as well as officials in the White House Faith Office and in the Department of Defense (Gabbatt, 2025; Graham, 2025). DRR and EC are not intrinsically oppositional, but the narratives and analogies of humanity, nature, and disaster currently emphasised in EC and evangelicals elevated to positions of power are clearly hostile to DRR. This is a significant challenge to advocates of DRR. Emphasising climate science as the final word on matters of DRR is not sufficient to address this matter, and may be a factor contributing to the divisiveness of DRR.

To be clear, the latest report from the IPCC warns, in no uncertain terms, that the contemporary climate crisis will increase extreme weather events and their associated economic and human costs (Pörtner et al., 2022). This assessment is supported by many forms of local and Indigenous knowledge, highlighting unprecedented changes in weather patterns and ecosystems. Tackling this climate crisis requires transformative changes in how societies prepare for and respond to disaster risk, as well as transformative changes in how societies incorporate alternative sources of knowledge, including EC. The internationally negotiated DRR framework aims to do just this, but is haunted both by its roots in liberal modernity and its silence on religious/spiritual beliefs. The most transformational programme of global social reordering to reduce human vulnerability to natural hazards was the shift to liberal modernity. Although this produced profound accomplishments and advancements across a wide range of human activity, it was an externally imposed, top‐down process based on physical and epistemic violence. It caused irreparable harm to many ecosystems, and created new vulnerabilities. The development of the global DRR framework differs substantially from the processes of colonisation and modernisation that forcefully spread liberal modernity around the world, but DRR remains strongly rooted in liberal, modernity. Successful incorporation of TILK that challenges liberal modernity is essential to facilitate the transformative potential of DRR.

EC, by contrast, is a fundamentally different challenge. To date, it has generated scepticism of climate science and opposed the expansion of global governance and state authority on DRR. Yet, insisting on the superiority of scientific/technical knowledge production and excluding EC and other religious interpretations of nature and disaster from the DRR framework has not succeeded in converting those who hold an enchanted view of nature. This paper argues that a pluriversal perspective is essential to overcome resistance to DRR and to identify valuable insights into humanity, nature, and disasters that are either absent from the liberal, modern framework, or understood through a different set of analogies and narratives.

EC perspectives on creation care, stewardship, and humanitarian relief represent significant zones of contact with scientific knowledge and TILK, and ought to be nurtured through meaningful engagement and inclusion. EC disaster narratives that emphasise punishment and/or conspiracy also cannot be ignored. The scapegoating and fatalist versions of these narratives ought to be consistently rejected and opposed through robust and transparent practices, while adopting a pluriversal inclusion viewpoint that requires assessing whether there is value in some version of these analogies. This assessment, I have argued, is based on whether they can co‐exist non‐hierarchically with other disaster analogies, and whether the analogy contributes to the preservation of healthy ecosystems on which human communities depend for their survival. The dominant punishment and conspiracy narratives common in EC are inconsistent with these values and thus have no worth in the discussion of DRR. In general terms, however, these disaster analogies may have merit by directing attention to moral dimensions of disaster and to the potential abuse of power, deception, and lack of transparency of the contemporary state‐based, neoliberal order.

## CONFLICT OF INTEREST STATEMENT

I declare no conflicts of interest.

## Data Availability

Data sharing not applicable to this article as no datasets were generated or analysed during the current study.

## Endnotes

^1^
This study understands disasters as socially constructed and accepts that the threshold and meaning of what constitutes a ‘disaster’ is determined by human actors and varies between and within societies. I use the term ‘disaster’ to describe significant disruptions to the existing social order caused by a combination of natural hazards and socially produced vulnerabilities. I use the term disaster narrative or analogy to capture how societies produce meaning associated with such disruptions, even if they do not employ the term disaster. Flood narratives and stories of famine, to take two common examples, are both understood as disaster narratives. I have not included social disruptions in which natural processes are not a proximate cause, such as wars or colonisation, which I capture through the terms physical and epistemic violence. I acknowledge that these distinctions are blurry in many cases, and that in many traditions, the language used to describe massive social disruption does not make such a distinction.

^2^
My appreciation to the peer reviewer who emphasised this point of tension and incompatibility.

^3^
Or deities, depending on the reading of Genesis and/or the interpretation of the Christian trinity of Father, Son, and Holy Spirit

^4^
For this paper, I reviewed the secondary literature on evangelical perspectives on disasters and the responses of major EC organisations and leaders in the US to Hurricanes Helene and Milton. I conducted website and Twitter (now ‘X') searches on key EC websites for the month‐long period between 26 September 2024 and 26 October 2024, including those of the National Association of Evangelicals, *Christianity Today*, Standing for Freedom Center, World Vision, Compassion International, Samaritan's Purse, and Habitat for Humanity. From these, I examined two documents that included a long‐form analysis of Evangelical narratives on disaster: one by William Wolfe (2024) at the Standing for Freedom Center (who served in the US Department of State under President Trump) and one by Russell Moore (2024) at *Christianity Today*.

## References

[disa70035-bib-0001] Adelman, S. (2018) ‘The Sustainable Development Goals, anthropocentrism and neoliberalism’. In D. French and L.J. Kotzé (eds.) Sustainable Development Goals: Law, Theory and Implementation. Edward Elgar Publishing, Chletenham. pp. 15–40.

[disa70035-bib-0002] Agensky, J.C. (2017) ‘Recognizing religion: politics, history, and the “long 19th century”’. European Journal of International Relations. 23(4). pp. 729–755.

[disa70035-bib-0003] Aitsi‐Selmi, A. , S. Egawa , H. Sasaki , C. Wannous , and V. Murray (2015) ‘The Sendai Framework for Disaster Risk Reduction: renewing the global commitment to people's resilience, health, and well‐being’. International Journal of Disaster Risk Science. 6(June). pp. 164–176.

[disa70035-bib-0004] Alberta, T. (2023) The Kingdom, the Power and the Glory: American Evangelicals in an Age of Extremis. Harper, New York City, NY.

[disa70035-bib-0005] Alexander, D.E. (2013) ‘Resilience and disaster risk reduction: an etymological journey’. Natural Hazards and Earth System Sciences. 13(11). pp. 2707–2716.

[disa70035-bib-0006] Attar, S. (2010) The Vital Roots of European Enlightenment: Ibn Tufayl's Influence on Modern Western Thought. Lexington Books, Lanham, MD.

[disa70035-bib-0007] Balay‐As, M. , J. Marlowe , and J‐C. Gaillard (2018) ‘Deconstructing the binary between indigenous and scientific knowledge in disaster risk reduction: approaches to high impact weather hazards’. International Journal of Disaster Risk Reduction. 30(Part A; September). pp. 18–24.

[disa70035-bib-0008] Barkun, M. (2011) Chasing Phantoms: Reality, Imagination, & Homeland Security Since 9/11. University of North Carolina Press, Chapel Hill, NC.

[disa70035-bib-0009] Barkun, M. (2013) A Culture of Conspiracy: Apocalyptic Visions in Contemporary America. University of California Press, Oakland, CA.

[disa70035-bib-0010] Barkun, M. (2015) ‘Conspiracy theories as stigmatized knowledge’. Diogenes. 62(3–4). pp. 114–120.

[disa70035-bib-0011] Barnett, M. and J.G. Stein (2012) Sacred Aid: Faith and Humanitarianism. Oxford University Press, Oxford.

[disa70035-bib-0012] Basham, L. (2019) ‘Malevolent global conspiracy’. In D. Coady (ed.) Conspiracy Theories: The Philosophical Debate. Routledge, Abingdon. pp. 93–105.

[disa70035-bib-0013] Beauchamp, J.D. (2022) ‘Evangelical identity and QAnon’. Journal of Religion and Violence. 10(1). pp. 17–36.

[disa70035-bib-0014] Bentz, L. (2013) ‘The top 10: Facebook “vomit” button for gays and other Pat Robertson quotes’. CNN (Cable News Network) website. 9 July. https://edition.cnn.com/2013/07/09/us/pat-robertson-facebook-remark (last accessed on 9 December 2025).

[disa70035-bib-0015] Berkes, F. (2012) Sacred Ecology. Routledge, Abingdon.

[disa70035-bib-0016] Berry, D. (2020) ‘Voting in the kingdom: prophecy voters, the New Apostolic Reformation, and Christian support for Trump’. Nova Religio: The Journal of Alternative and Emergent Religions. 23(4). pp. 69–93.

[disa70035-bib-0017] Blaikie, P. , T. Cannon , I. Davis , and B. Wisner (1994) At Risk: Natural Hazards, *People's Vulnerability and Disasters* . Routledge, Abingdon.

[disa70035-bib-0018] Blaser, M. (2013) ‘Ontological conflicts and the stories of peoples in spite of Europe: towards a conversation on political ontology’. Current Anthropology. 54(5). pp. 547–568.

[disa70035-bib-0019] Blewitt, J. (2008) Understanding Sustainable Development. Routledge, Abingdon.

[disa70035-bib-0020] Boorse, D. (2022) Loving the Least of These: Addressing a Changing Environment. National Association of Evangelicals, Washington, DC.

[disa70035-bib-0021] Borenstein, S. (2022) ‘UN climate report: “atlas of human suffering” worse, bigger’. AP (Associated Press) website. 28 February. https://apnews.com/article/climate-science-europe-united-nations-weather-8d5e277660f7125ffdab7a833d9856a3 (last accessed on 9 December 2025).

[disa70035-bib-0022] Borrows, J. (2018) ‘Earth‐bound: indigenous resurgence and environmental reconciliation’. In M. Asch , J. Borrows , and J. Tully (eds.) Resurgence and Reconciliation: Indigenous–Settler Relations and Earth Teachings. University of Toronto Press, Toronto, ON. pp. 49–82.

[disa70035-bib-0023] Börzel, T.A. and M. Zürn (2021) ‘Contestations of the liberal international order: from liberal multilateralism to postnational liberalism’. International Organization. 75(2). pp. 282–305.

[disa70035-bib-0024] Butler, K. (2024) ‘God or deep state? Christian nationalists can't decide who to blame for Hurricane Helene’. Mother Jones website. 8 October. https://www.motherjones.com/politics/2024/10/god-or-deep-state-christian-nationalists-cant-decide-who-to-blame-for-hurricane-helene/ (last accessed on 9 December 2025).

[disa70035-bib-0025] Calhoun, C. , M. Juergensmeyer , and J. VanAntwerpen (2011) Rethinking Secularism. Oxford University Press, New York City, NY.

[disa70035-bib-0026] Cannon, T. (1994) ‘Vulnerability analysis and the explanation of “natural” disasters’. In A. Varley (ed.) Disasters, Development and Environment. John Wiley & Sons, Chichester. pp. 13–30.

[disa70035-bib-0027] Carrigan, A. (2015) ‘Towards a postcolonial disaster studies’. In E. DeLoughrey , J. Didur , and A. Carrigan (eds.) Global Ecologies and the Environmental Humanities: Postcolonial Approaches. Routledge, New York City, NY. pp. 116–139.

[disa70035-bib-0028] Casanova, J. (2009) ‘The secular and secularisms’. Social Research. 76(4). pp. 1049–1066.

[disa70035-bib-0029] Chakrabarty, D. (2007) ‘History and the politics of recognition’. In S. Morgan , K. Jenkins , and A. Munslow (eds.) Manifestos for History. Routledge, Abingdon. pp. 77–87.

[disa70035-bib-0030] Chester, D.K. (1998) ‘The theodicy of natural disasters’. Scottish Journal of Theology. 51(4). pp. 485–506.

[disa70035-bib-0031] Chester, D.K. (2005) ‘Theology and disaster studies: the need for dialogue’. Journal of Volcanology and Geothermal Research. 146(4). pp. 319–328.

[disa70035-bib-0032] Chester, D.K. and A.M. Duncan (2010) ‘Responding to disasters within the Christian tradition, with reference to volcanic eruptions and earthquakes’. Religion. 40(2). pp. 85–95.

[disa70035-bib-0033] Coady, D. (2019) ‘Conspiracy theories and official stories’. In D. Coady (ed.) Conspiracy Theories: The Philosophical Debate. Routledge, Abingdon. pp. 115–127.

[disa70035-bib-0034] Cohn, N. (1970) The Pursuit of the Millennium. Oxford University Press, Oxford.

[disa70035-bib-0035] Connolly, W.E. (2005) Pluralism. Duke University Press, Durham, NC.

[disa70035-bib-0036] Conway, P.R. (2025) ‘Repressive suspicion, or: the problem with conspiracy theories’. Cultural Studies. 39(4). pp. 514–545.

[disa70035-bib-0037] Cox, J. , G. Finau , R. Kant , J. Tarai , and J. Titifanue (2018) ‘Disaster, divine judgment, and original sin: Christian interpretations of Tropical Cyclone Winston and climate change in Fiji’. The Contemporary Pacific. 30(2). pp. 380–411.

[disa70035-bib-0038] Cutter, S. (1996) ‘Vulnerability to environmental hazards’. Progress in Human Geography. 20(4). pp. 529–539.

[disa70035-bib-0039] de Sousa Santos, B. (2015) Epistemologies of the South: Justice Against Epistemicide. Routledge, Abingdon.

[disa70035-bib-0040] Du Pisani, J.A. (2006) ‘Sustainable development – historical roots of the concept’. Environmental Sciences. 3(2). pp. 83–96.

[disa70035-bib-0041] Dynes, R.R. (1993) ‘Disaster reduction: the importance of adequate assumptions about social organization’. Sociological Spectrum. 13(1). pp. 175–192.

[disa70035-bib-0042] Ensor, M.O. (2003) ‘Disaster evangelism: religion as a catalyst for change in post‐Mitch Honduras’. International Journal of Mass Emergencies & Disasters. 21(2). pp. 31–49.

[disa70035-bib-0043] Eom, K. and S.T. Ng (2023) ‘The potential of religion for promoting sustainability: the role of stewardship’. TopiCS: Topics in Cognitive Science. 15(3). pp. 480–499. 36780334 10.1111/tops.12641

[disa70035-bib-0044] Escobar, A. (2018) Designs for the Pluriverse: Radical Interdependence, Autonomy, and the Making of Worlds. Duke University Press, Durham, NC.

[disa70035-bib-0045] Falk, R.A. (1972) This Endangered Planet: Prospects and Proposals for Human Survival. Vintage Books, New York City, NY.

[disa70035-bib-0046] Feldman, A. and A. Ohnsman (2024) ‘Misinformation and conspiracy theories hamper Hurricane Relief’. *Forbes* website. 14 October. https://www.forbes.com/sites/amyfeldman/2024/10/10/misinformation-and-conspiracy-theories-hamper-hurricane-relief-ev-batteries-recycling-lego-sustainable/ (last accessed on 9 December 2025).

[disa70035-bib-0047] FEMA (Federal Emergency Management Agency) (2024) ‘Hurricane rumor response’. Website. 13 October. https://www.fema.gov/disaster/recover/rumor/hurricane-rumor-response (last accessed on 9 December 2025).

[disa70035-bib-0048] FEMA (2025) *FEMA Ends Wasteful, Politicized Grant Program, Returning Agency to Core Mission of Helping Americans Recovering from Natural Disasters*. Advisory. 4 April. FEMA, Washington, DC.

[disa70035-bib-0049] Fitzgerald, F. (2018) The Evangelicals: The Struggle to Shape America. Simon & Schuster. New York City, NY.

[disa70035-bib-0050] Fountain, P.M. , S.L. Kindon , and W.E. Murray (2004) ‘Christianity, calamity, and culture: the involvement of Christian churches in the 1998 Aitape tsunami disaster relief’. The Contemporary Pacific. 16(2). pp. 321–355.

[disa70035-bib-0051] Gabbatt, A. (2025) ‘Trump has put Christian nationalists in key roles – say a prayer for free speech’. *The Guardian* website. 16 April. https://www.theguardian.com/us-news/2025/apr/16/christian-nationalists-trump-administration (last accessed on 10 December 2025).

[disa70035-bib-0052] Gaillard, J‐C. (2019) ‘Disaster studies inside out’. Disasters. 43(S1). pp. S7–S17. 30575089 10.1111/disa.12323

[disa70035-bib-0053] Gaillard, J‐C. (2021) The Invention of Disaster: Power and Knowledge in Discourses on Hazard and Vulnerability. Routledge, Abingdon.

[disa70035-bib-0054] Gaillard, J‐C. and J. Mercer (2013) ‘From knowledge to action: bridging gaps in disaster risk reduction’. Progress in Human Geography. 37(1). pp. 93–114.

[disa70035-bib-0055] Gaillard, J‐C. and P. Texier (2010) ‘Religions, natural hazards, and disasters: an introduction’. Religion. 40(2). pp. 81–84.

[disa70035-bib-0056] Graham, R. (2025) ‘Christianity in the White House’. *The New York Times* website. 18 April. https://www.nytimes.com/2025/04/18/briefing/christianity-in-the-white-house.html (last accessed on 10 December 2025).

[disa70035-bib-0057] Grandjean, D. , A‐C. Rendu , T. MacNamee , and K.R. Scherer (2008) ‘The wrath of the gods: appraising the meaning of disaster’. Social Science Information. 47(2). pp. 187–204.

[disa70035-bib-0058] Green, D. and G. Raygorodetsky (2010) ‘Indigenous knowledge of a changing climate’. Climate Change. 100(January). pp. 239–242.

[disa70035-bib-0059] Gribben, C. (2021) Survival and Resistance in Evangelical America: Christian Reconstruction in the Pacific Northwest. Oxford University Press, New York City, NY.

[disa70035-bib-0060] Grosfoguel, R. (2013a) ‘The epistemic decolonial turn: beyond political‐economy paradigms’. In W.D. Mignolo and A. Escobar (eds.) Globalization and the Decolonial Option. Routledge, Abingdon. pp. 65–77.

[disa70035-bib-0061] Grosfoguel, R. , (2013b) ‘The structure of knowledge in Westernized universities’. Human Architecture: Journal of the Sociology of Self‐Knowledge. 11(1). pp. 73–90.

[disa70035-bib-0062] Guadagno, L. (2016) ‘Human mobility in the Sendai Framework for Disaster Risk Reduction’. International Journal of Disaster Risk Science. 7(March). pp. 30–40.

[disa70035-bib-0063] Hadlos, A. , A. Opdyke , and S.A. Hadigheh (2022) ‘Where does local and indigenous knowledge in disaster risk reduction go from here? A systematic literature review’. International Journal of Disaster Risk Reduction. 79(September). Article number: 103160. 10.1016/j.ijdrr.2022.103160.

[disa70035-bib-0064] Hannigan, J. (2012) Disasters Without Borders: The International Politics of Natural Disasters. Polity Press, Cambridge.

[disa70035-bib-0065] Harding, A. (2018) ‘One planet, many societies’. In B. Reiter (ed.) Constructing the Pluriverse: The Geopolitics of Knowledge. Duke University Press, Durham, NC. pp. 39–62.

[disa70035-bib-0066] Harding, S. (2008) Sciences from Below: Feminisms, Postcolonialities, and Modernities. Duke University Press, Durham, NC.

[disa70035-bib-0067] Henry, J. (1997) The Scientific Revolution and the Origins of Modern Science. St. Martin's Press, New York City, NY.

[disa70035-bib-0068] Hewitt, K. (1983) Interpretation of Calamities: From the Viewpoint of Human Ecology. Allen & Unwin, Winchester, MA.

[disa70035-bib-0069] Hewitt, K. (2009) *The Social Nature of Exposure, Vulnerability and Responses to Disaster*. Paper prepared for the ‘International Workshop on Glacier and Permafrost Hazards in Mountains’, Vienna, Austria, 10–13 November 2009. https://boku.ac.at/fileadmin/data/H03000/H87000/H87200/4Veranstaltungen/Hewitt.pdf (last accessed on 10 December 2025).

[disa70035-bib-0070] Hilhorst, D. , J. Baart , G. van der Haar , and F.M. Leeftink (2015) ‘Is disaster “normal” for indigenous people? Indigenous knowledge and coping practices’. Disaster Prevention and Management. 24(4). pp. 506–522.

[disa70035-bib-0071] Hoffman, S.M. and A. Oliver‐Smith (2019) ‘Introduction to the second edition of *The Angry Earth*: from introduction to widespread reception’. In A. Oliver‐Smith and S.M. Hoffman (eds.) The Angry Earth: Disaster in Anthropological Perspective. Routledge, Abingdon. pp. 1–14.

[disa70035-bib-0072] Hollis, S. (2020) Resilience in the Pacific and the Caribbean: The Local Construction of Disaster Risk Reduction. Routledge, Abingdon.

[disa70035-bib-0073] Hulme, M. (2014) ‘Attributing weather extremes to “climate change”: a review’. Progress in Physical Geography. 38(4). pp. 499–511.

[disa70035-bib-0074] Hutchings, K. (2019) ‘Decolonizing global ethics: thinking with the pluriverse’. Ethics & International Affairs. 33(2). pp. 115–125.

[disa70035-bib-0075] Jacobsen, S.G. (2013) ‘Chinese influences or images? Fluctuating histories of how Enlightenment Europe read China’. Journal of World History. 24(3). pp. 623–660.

[disa70035-bib-0076] James, P. and M.B. Steger (2023) ‘Rethinking pluriversal theory in globalization research: bringing ontology in’. Global Perspectives. 4(1). Article number: 88513. 10.1525/gp.2023.88513.

[disa70035-bib-0077] James, W. (1977) A Pluralistic Universe. Harvard University Press, Cambridge, MA.

[disa70035-bib-0078] Joseph, J. (2013) ‘Resilience as embedded neoliberalism: a governmentality approach’. Resilience: International Policies, Practices and Discourses. 1(1). pp. 38–52.

[disa70035-bib-0079] Kaell, H. (2022) ‘American theodicy: human nature and natural disaster’. In J. Bruner and D.C. Kirkpatrick (eds.) Global Visions of Violence: Agency and Persecution in World Christianity. Rutgers University Press, New Brunswick, NJ. pp. 76–102.

[disa70035-bib-0080] Keane, J. (2000) ‘Secularism?’. The Political Quarterly. 71(S1). pp. 5–19.

[disa70035-bib-0081] Kelman, I. (2020) Disaster by Choice: How Our Actions Turn Natural Hazards into Catastrophes. Oxford University Press, Oxford.

[disa70035-bib-0082] Kelman, I. , J. Mercer, and J.‐C. Gaillard (2012) 'Indigenous knowledge and disaster risk reduction'. Geography. 97(1). pp.12–21

[disa70035-bib-0083] Khamrang Varah, S. , E. Khongrei , M. Mahongnao , and F. Varah (2021) ‘The influence of religion on beliefs of stewardship, dominionship and controlling god towards pro‐environmental support’. Culture and Religion. 22(1). pp. 84–101.

[disa70035-bib-0084] Klein, N. (2007) The Shock Doctrine: The Rise of Disaster Capitalism. Macmillan, New York City, NY.

[disa70035-bib-0085] Kölbl‐Ebert, M. (2009) Geology and Religion: A History of Harmony and Hostility. Geological Society of London, London.

[disa70035-bib-0086] Ladd, K.L. and B. Spilka (2002) ‘Inward, outward, and upward: cognitive aspects of prayer’. Journal for the Scientific Study of Religion. 41(3). pp. 475–484.

[disa70035-bib-0087] Loewenstein, A. (2015) Disaster Capitalism: Making a Killing Out of Catastrophe. Verso Books, London.

[disa70035-bib-0088] Löfflmann, G. (2022) ‘Introduction to special issue: the study of populism in international relations’. The British Journal of Politics and International Relations. 24(3). pp. 403–415.

[disa70035-bib-0089] Lowe, B.S. et al. (2022) ‘The generational divide over climate change among American evangelicals’. Environmental Research Letters. 17(11). Article number: 114020. 10.1088/1748-9326/ac9a60.

[disa70035-bib-0090] Lynch, C. (2020) Wrestling with God: Ethical Precarity in Christianity and International Relations. Cambridge University Press, Cambridge.

[disa70035-bib-0091] Lynch, C. and T.B. Schwarz (2016) ‘Humanitarianism's proselytism problem’. International Studies Quarterly. 60(4). pp. 636–646.

[disa70035-bib-0092] M.R . (2012) ‘Punish them for their sins’. *The Economist* website. 7 November. https://www.economist.com/pomegranate/2012/11/07/punish-them-for-their-sins (last accessed on 9 December 2025).

[disa70035-bib-0093] MacMillen, S.L. and T. Rush (2022) ‘QAnon—religious roots, religious responses’. Critical Sociology. 48(6). pp. 989–1004.

[disa70035-bib-0094] Martin, D. (2017) On Secularization: Towards a Revised General Theory. Routledge, Abingdon.

[disa70035-bib-0095] Massey, K. and J. Sutton (2007) ‘Faith community's role in responding to disasters’. Southern Medical Journal. 100(9). pp. 944–945. 17902313 10.1097/SMJ.0b013e318145a847

[disa70035-bib-0096] McEntire, D.A. (2004) ‘Tenets of vulnerability: an assessment of a fundamental disaster concept’. Journal of Emergency Management. 2(2). pp. 23–29.

[disa70035-bib-0097] McGeehan, K.M. and C.K. Baker (2017) ‘Religious narratives and their implications for disaster risk reduction’. Disasters. 41(2). pp. 258–281. 27237944 10.1111/disa.12200

[disa70035-bib-0098] McLeod, L.J. et al. (2024) ‘Environmental stewardship: a systematic scoping review’. PLoS ONE. 19(5). Article number: e0284255. 10.1371/journal.pone.0284255. 38713707 PMC11075856

[disa70035-bib-0099] Meyer, J.W. , J. Boli , G. Thomas , and F.O. Ramirez (1997) ‘World society and the nation‐state’. American Journal of Sociology. 103(1). pp. 144–181.

[disa70035-bib-0100] Mignolo, W.D. (2018a) ‘On pluriversality and multipolar world order: decoloniality after decolonization; dewesternization after the Cold War’. In B. Reiter (ed.) Constructing the Pluriverse: The Geopolitics of Knowledge. Duke University Press, Durham, NC. pp. 90–116.

[disa70035-bib-0101] Mignolo, W.D. (2018b) ‘Foreword: on pluriversality and multipolarity’. In B. Reiter (ed.) Constructing the Pluriverse: The Geopolitics of Knowledge. Duke University Press, Durham, NC. pp. x–xiv.

[disa70035-bib-0102] Millman, O. (2024) “It's mindblowing: US meteorologists face death threats as hurricane conspiracies surge’. *The Guardian* website. 11 October. https://www.theguardian.com/us-news/2024/oct/11/meteorologists-death-threats-hurricane-conspiracies-misinformation (last accessed on 11 December 2025).

[disa70035-bib-0103] Mitchell, J.T. (2003) ‘Prayer in disaster: case study of Christian clergy’. Natural Hazards Review. 4(1). pp. 20–26.

[disa70035-bib-0104] Moore, R. (2024) ‘A hurricane doesn't tell us who to hate’. *Christianity Today* website. 2 October. https://www.christianitytoday.com/2024/10/hurricane-helene-hate-social-media-russell-moore/ (last accessed on 11 December 2025).

[disa70035-bib-0105] Moreno, I. (2025) ‘Trump's cuts to FEMA leave us unprepared for disasters’. NRDC (Natural Resources Defense Council) website. 24 February. https://www.nrdc.org/media/trumps-cuts-fema-leave-us-unprepared-disasters (last accessed on 11 December 2025).

[disa70035-bib-0106] Muppidi, H. (2004) ‘Colonial and postcolonial governance’. In M. Barnett and R. Duvall (eds.) Power in Global Governance. Cambridge University Press, Cambridge. pp. 273–293.

[disa70035-bib-0107] Njus, D.M. and A. Scharmer (2020) ‘Evidence that God attachment makes a unique contribution to psychological well‐being’. The International Journal for the Psychology of Religion. 30(3). pp. 178–201.

[disa70035-bib-0108] O'Mathúna, D.P. (2018) ‘Christian theology and disasters: where is God in all this?’. In D.P. O'Mathúna , V. Dranseika , and B. Gordijn (eds.) Disasters: Core Concepts and Ethical Theories. Springer Nature, Cham. pp. 27–42.

[disa70035-bib-0109] Oliver‐Smith, A. (2016) ‘Disaster risk reduction and applied anthropology’. Annals of Anthropological Practice. 40(1). pp. 73–85.

[disa70035-bib-0110] Oslender, U. (2018) ‘Local aquatic epistemologies among black communities on Columbia's Pacific Coast and the pluriverse’. In B. Reiter (ed.) Constructing the Pluriverse: The Geopolitics of Knowledge. Duke University Press, Durham, NC. pp. 137–150.

[disa70035-bib-0111] Paice, E. (2008) Wrath of God: The Great Lisbon Earthquake of 1755. Quercus, London.

[disa70035-bib-0112] Pally, M. (2022) White Evangelicals and Right‐Wing Populism: How Did We Get Here? Routledge, Abingdon.

[disa70035-bib-0113] Parasram, V. (2018) ‘Hunting the state of nature: race and ethics in postcolonial international relations’. In B.J. Steele and E. Heinze (eds.) Routledge Handbook of Ethics and International Relations. Routledge, Abingdon. pp. 102–155.

[disa70035-bib-0114] Patterson, J.A. (1988) ‘Changing images of the beast: apocalyptic conspiracy theories in American history’. Journal of the Evangelical Theological Society. 31(4). pp. 443–452.

[disa70035-bib-0115] Pelling, M. (2003) ‘Paradigms of risk’. In M. Pelling (ed.) Natural Disaster and Development in a Globalizing World. Routledge, Abingdon. pp. 3–16.

[disa70035-bib-0116] Platt, R. (1999) Disasters and Democracy: The Politics of Extreme Natural Events. Island Press, Washington, DC.

[disa70035-bib-0117] Pogue, N. (2024) ‘Winning against God's earth: the Religious Right's use of a competition framework to justify anti‐environmentalism’. The Journal of American Culture. 47(1). pp. 50–56.

[disa70035-bib-0118] Pörtner, H‐O. et al. (2022) 'Climate Change 2022: Impacts, Adaptation, and Vulnerability'. Working Group II Contribution to the Sixth Assessment Report of the Intergovernmental Panel on Climate Change. Summary for Policymakers. Cambridge University Press, Cambridge.

[disa70035-bib-0119] Reid, J. (2012) ‘The disastrous and politically debased subject of resilience’. Development Dialogue. 58(April). pp. 67–79.

[disa70035-bib-0120] Reiter, B. (2018) ‘Introduction’. In B. Reiter (ed.) Constructing the Pluriverse: The Geopolitics of Knowledge. Duke University Press, Durham, NC. pp. 1–17.

[disa70035-bib-0121] Rozario, K. (2019) The Culture of Calamity: Disaster & the Making of Modern America. University of Chicago Press, Chicago, IL.

[disa70035-bib-0122] Rudiak‐Gould, P. (2014) ‘Climate change and accusation: global warming and local blame in a small island state’. Current Anthropology. 55(4). pp. 365–386.

[disa70035-bib-0123] Savransky, M. (2021) Around the Day in Eighty Worlds: Politics of the Pluriverse. Duke University Press, Durham, NC.

[disa70035-bib-0124] Schaarsberg, S.K. (2024) ‘Enacting the pluriverse in the West: contemplative activism as a challenge to the disenchanted one‐world world’. European Journal of International Relations. 30(2). pp. 434–460.

[disa70035-bib-0125] Schipper, E.L. (2010) 'Religion as an integral part of determining and reducing climate change and disaster risk: an agenda for research’. In M. Voss (ed.) Der Klimawandel. VS Verlag für Sozialwissenschaften, Wiesbaden. pp. 377–393.

[disa70035-bib-0126] Schmidt, J. (2014) ‘Intuitively neoliberal? Towards a critical understanding of resilience governance’. European Journal of International Relations. 21(2). pp. 402–426.

[disa70035-bib-0127] Scott, J.C. (1998) Seeing Like a State: How Certain Schemes to Improve the Human Condition Have Failed. Yale University Press, New Haven, CT.

[disa70035-bib-0128] Shimoyama, S. (2002) ‘Basic characteristics of disaster’. In J. Grattan and Robin Torrence (eds). Natural Disasters and Cultural Change. Routledge, Abingdon. pp. 19–27.

[disa70035-bib-0129] Shrady, N. (2008) The Last Day: Wrath, Ruin, and Reason in the Great Lisbon Earthquake of 1755. Viking, New York City, NY.

[disa70035-bib-0130] Sims, J.H. and D.D. Baumann (1972) ‘The tornado threat: coping styles of the North and South: tornado death rates in Illinois and Alabama may be related to the psychology of their residents’. Science. 176(4042). pp. 1386–1392. 17834637 10.1126/science.176.4042.1386

[disa70035-bib-0131] Slobodian, Q. (2025) Hayek's Bastards: Race, Gold, IQ, and the Capitalism of the Far Right. Princeton University Press, Princeton, NJ.

[disa70035-bib-0132] Spector, M. and J.I. Kitsuse (1987) Constructing Social Problems. Transaction Publishers, Piscataway, NJ.

[disa70035-bib-0133] Spilka, B. and K.L. Ladd (2012) The Psychology of Prayer: A Scientific Approach. Guilford Press, New York City, NY.

[disa70035-bib-0134] Starblanket, G. and H. Kiiwetinepinesiik Stark (2018) ‘Towards a relational paradigm – four points for consideration: knowledge, gender, land, and modernity’. In M. Asch , J. Borrows , and J. Tully (eds.) Resurgence and Reconciliation: Indigenous–Settler Relations and Earth Teachings. University of Toronto Press, Toronto, ON. pp. 175–208.

[disa70035-bib-0135] Steinberg, T. (2006) Acts of God: The Unnatural History of Natural Disaster in America. Oxford University Press, New York City, NY.

[disa70035-bib-0136] Thompson, S. (2024) ‘How election deniers sank a security conference in Georgia’. *The New York Times* website. 1 November. https://www.nytimes.com/2024/11/01/technology/election-deniers-georgia.html (last accessed on 11 December 2025).

[disa70035-bib-0137] Thunström, L. and S. Noy (2019) ‘The value of thoughts and prayers’. Proceedings of the National Academy of Sciences of the United States of America. 116(40). pp. 19797–19798. 31527228 10.1073/pnas.1908268116 PMC6778220

[disa70035-bib-0138] Tierney, K.J. (2007) ‘From the margins to the mainstream? Disaster research at the crossroads’. Annual Review of Sociology. 33(August). pp. 503–525.

[disa70035-bib-0139] Tierney, K.J. (2015) ‘Resilience and the neoliberal project: discourses, critiques, practices—and Katrina’. American Behavioral Scientist. 59(10). pp. 1327–1342.

[disa70035-bib-0140] Tozier de La Poterie, A. and M‐A. Baudoin (2015) ‘From Yokohama to Sendai: approaches to participation in international disaster risk reduction frameworks’. International Journal of Disaster Risk Science. 6(June). pp. 128–139.

[disa70035-bib-0141] Tully, J. (1995) Strange Multiplicity: Constitutionalism in an Age of Diversity. Cambridge University Press, Cambridge.

[disa70035-bib-0142] Turner, N.J. and P. Spalding (2018) ‘Learning from the Earth, learning from each other: ethnoecology, responsibility, and reciprocity’. In M. Asch , J. Borrows , and J. Tully (eds.) Resurgence and Reconciliation: Indigenous–Settler Relations and Earth Teachings. University of Toronto Press, Toronto, ON. pp. 265–292.

[disa70035-bib-0143] Udías, A. (2009) ‘Earthquakes as God's punishment in 17th‐ and 18th‐century Spain’. In M. Kölbl‐Ebert (ed.) Geology and Religion: A History of Harmony and Hostility. Geological Society of London, London. pp. 41–48.

[disa70035-bib-0144] UNISDR (United Nations Office for Disaster Risk Reduction) (2015) Sendai Framework for Disaster Risk Reduction 2015–2030. UNISDR, Geneva.

[disa70035-bib-0145] Uscinski, J.E. (2018) ‘The study of conspiracy theories’. Argumenta. 3(2). pp. 233–245.

[disa70035-bib-0146] van Eck Duymaer van Twist, A. and S. Newcombe (2018) ‘Trust me, you can't trust them: stigmatized knowledge in cults and conspiracies’. In A. Dyrendal , D.G. Robertson , and E. Asprem (eds.) Handbook of Conspiracy Theory and Contemporary Religion. Brill, Leiden. pp. 152–179.

[disa70035-bib-0147] van Kersbergen, K. (2003) Social Capitalism: A Study of Christian Democracy and the Welfare State. Routledge, London.

[disa70035-bib-0148] Vanderford, R. (2025) ‘Natural disasters cost $417 billion worldwide in 2024’. *The Wall Street Journal* website. 22 January. https://www.wsj.com/articles/natural‐disasters‐cost‐417‐billion‐worldwide‐in‐2024‐1bf513f3?gaa_at=eafs&gaa_n=AWEtsqdC8xMf0t0KWPbkS815G2Q9pr_9X0RIn1Ib1fn7K2B‐ULm54YEN9Al1GIQ7lwU%3D&gaa_ts=693bea3e&gaa_sig=jP_8UHLQhk7XzWHEyMWZApFXupOXImeipbr39jYTpqLYxYJBjM3‐d1pqH3Y_xQ3KQf6DyNMhPTER8D_FRKVLCg%3D%3D (last accessed on 11 December 2025).

[disa70035-bib-0149] Varley, A. (1994) Disasters, Development and Environment. J. Wiley & Sons, New York City, NY.

[disa70035-bib-0150] Veldman, R.G. , D.M. Wald , S.B. Mills , and D.A. Peterson (2021) ‘Who are American evangelical protestants and why do they matter for US climate policy?’. WIREs: Climate Change. 12(2). Article number: e693. 10.1002/wcc.693.

[disa70035-bib-0151] von Wright, G.H. (1997) ‘Progress: fact and fiction’. The A. Burgen , P. McLaughlin , and J. Mittelstraß (eds.) Idea of Progress. De Gruyter, Berlin. pp. 1–18.

[disa70035-bib-0152] Wajner, D.F. (2023) *Delegitimizing International Institutions: How Global Populism Challenges the Liberal International Order*. SCRIPTS Working Paper No. 30. https://www.scripts-berlin.eu/publications/working-paper-series/Working-Paper-30-2023/index.html (last accessed on 11 December 2025).

[disa70035-bib-0153] Walker, J. (2015) ‘God, gays, and voodoo: voicing blame after Katrina’. Communication and Theater Association of Minnesota Journal. 41(1). pp. 29–48.

[disa70035-bib-0154] Walker, V. (2024) ‘Even amid natural disaster, God is sovereign’. Standing for Freedom Center website. 9 October. https://www.standingforfreedom.com/2024/10/09/even-amid-natural-disaster-god-is-sovereign/ (last accessed on 11 December 2025).

[disa70035-bib-0155] Walter, S. (2021) ‘The backlash against globalization’. Annual Review of Political Science. 24(1). pp. 421–442.

[disa70035-bib-0156] White, R.S. (2016) ‘Disasters, nature, and acts of God’. In G. Buxton , N.C. Habel , and D. Rhoads (eds.) The Nature of Things: Rediscovering the Spiritual in God's Creation. Pickwick Publications, Eugene, OR. pp. 103–116.

[disa70035-bib-0157] Wisner, B. (2003) ‘Changes in capitalism and global shifts in the distribution of hazard and vulnerability’. In M. Pelling (ed.) Natural Disaster and Development in a Globalizing World. Routledge, Abingdon. pp. 43–56.

[disa70035-bib-0158] WMO (World Meteorological Organization) (2025) *State of the Global Climate 2024*. WMO‐No. 1368. March. https://wmo.int/publication-series/state-of-global-climate-2024 (last accessed on 11 December 2025).

[disa70035-bib-0159] Wolfe, W. (2024) ‘Tracing the hand of God through the storm’. Standing for Freedom Center website. 10 October. https://www.standingforfreedom.com/2024/10/tracing-the-hand-of-god-through-the-storm/ (last accessed on 11 December 2025).

[disa70035-bib-0160] Wood, M. and K. Douglas (2018) ‘Are conspiracy theories a surrogate for God?’. In A. Dyrendal , D.G. Robertson , and E. Asprem (eds.) Handbook of Conspiracy Theory and Contemporary Religion. Brill, Leiden. pp. 87–105.

[disa70035-bib-0161] Worthen, M. (2014) Apostles of Reason: The Crisis of Authority in American Evangelicalism. Oxford University Press, Oxford.

[disa70035-bib-0162] Yilmaz, I. , N. Morieson , and M. Demir (2021) ‘Exploring religions in relation to populism: a tour around the world’. Religions. 12(5). Article number: 301. 10.3390/rel12050301.

[disa70035-bib-0163] Zajac, A. (2010) ‘Haiti earthquake: televangelist Pat Robertson links quake to “pact with the devil”’. *Los Angeles Times* website. 13 January. https://www.latimes.com/archives/la-xpm-2010-jan-13-la-fg-haiti-robertson14-2010jan14-story.html (last accessed on 11 December 2025).

